# Improvement of left ventricular ejection fraction in revascularized postmyocardial patients: indication for statistical fallacy

**DOI:** 10.1186/s13104-017-2562-4

**Published:** 2017-07-05

**Authors:** Rona Reibis, Annett Salzwedel, Klaus Bonaventura, Heinz Völler, Karl Wegscheider

**Affiliations:** 1 Cardiological Outpatient Clinic Am Park Sanssouci, Potsdam, Germany; 2 Center of Rehabilitation Research, University of Potsdam, Am Neuen Palais 10, Haus 12, 14469 Potsdam, Germany; 3 Department of Cardiology and Angiology, Ernst von Bergmann Hospital, Potsdam, Germany; 4 Department of Cardiology, Klinik am See, Rehabilitation Center of Cardiovascular Diseases, Ruedersdorf, Berlin, Germany; 5 Department of Medical Biometry and Epidemiology, University Medical Center Hamburg-Eppendorf, Hamburg, Germany

**Keywords:** Myocardial infarction, Heart failure, Cardioverter-defibrillator, Regression toward the mean

## Abstract

**Background:**

Reduced left ventricular ejection fraction (LVEF) ≤30% is the most powerful prognostic indicator for sudden cardiac death (SCD) in patients after myocardial infarction (MI), but there are little data about long-term changes of LVEF after revascularization and the following implantation of a cardioverter defibrillator (ICD).

**Methods:**

We performed a retrospective analysis of 277 patients with reduced LVEF at least 1 month after MI and complete revascularization. Patients (median time post-MI 23.4 months; 74.3% after PCI, 25.7% after CABG were assigned either to group 1 (LVEF <30%) or group 2 (LVEF 30–40%). Biplane echocardiography was redone after a mean follow-up of 441 ± 220 days.

**Results:**

LVEF increased significantly in both two groups (group 1: 26.2 ± 4.8% to 32.4 ± 8.5%; *p* < 0.001; group 2: 38.2 ± 2.5% to 44.4 ± 9.6%; *p* < 0.001). However, statistical analysis of first and second LVEF measurement by means of a LOWESS regression and with an appropriate correction of the regression towards the mean effect revealed only a moderate increase of the mean LVEF from 35 to 37% (p < 0.001) with a large interindividual variation.

**Conclusions:**

The impact of early revascularization on LVEF appears to be low in the majority of post-MI heart failure patients. Owing to the high variability, a single measurement may not be reliable enough to justify a decision on ICD indication.

## Background

While not consistently used across studies, the terms sudden cardiac arrest and sudden cardiac death (SCD) describe the unexpected abrupt circulatory arrest, typically owing to sustained ventricular tachycardia/ventricular fibrillation [1, 2]. These events commonly occur in patients with structural heart disease, in particular coronary heart disease (CHD) including myocardial infarction (MI).

Severely depressed left ventricular ejection fraction (LVEF) of less than 30% is the most powerful indicator for life-threatening ventricular tachyarrhythmias and SCD in patients after MI. Implantation of a cardioverter defibrillator (ICD) effectively improves survival in this high-risk population [3–5]. Although additional risk factors were also shown to be of predictive value [6], the estimation of SCD risk in the present study is based on the left ventricular ejection fraction. However, how the LVEF following MI changes over time is unknown. Conflicting data have been published concerning the time of ICD implantation [7–11]. It is not certain in how many cases LVEF improves over time and to what extent, especially in patients with stunned or hibernating myocardium [12–14]. Serial echocardiography is often used in clinical studies and but also in daily cardiological practice to evaluate left ventricular systolic function after MI [15, 16]. However, the reproducibility of echocardiographically determined LVEF may be poor [17, 18]. LVEF serial measurements are limited by a high interindividual as well as intraindividual variability [19–22] that is only partially controlled by 3D volumetry and optimized endocardial border detection by contrast echocardiography [23, 24].

Against this background, we systematically studied the LVEF changes in revascularized post-MI heart failure patients with an LVEF of <40% in order to give recommendations for LVEF measurements before ICD implantation to prevent SCD.

## Methods

### Design

This was a retrospective analysis of the prospective multi-center German PreSCD (Prevention of sudden cardiac death)-II-registry which enrolled 10,530 post-MI patients.

### Patients

In the context of the registry, between November 2003 and October 2005, a total of 2046 patients at least 1 month after documented MI (median 1.2 months) were included in the monocentric substudy. All patients were hospitalized for cardiological rehabilitation in the Cardiovascular Rehabilitation Center Rüdersdorf, Germany.

Physical capacity during symptom-limited bicycle exercise ECG as well as LVEF quantified echocardiographically were documented. Out of the 2046 patients, 277 consecutive patients with LVEF ≤40% were selected for further follow-up. Two strata were prospectively defined depending on whether LVEF was <30% or 30–40%, respectively.

### LVEF measurements

**Fig. 1:**
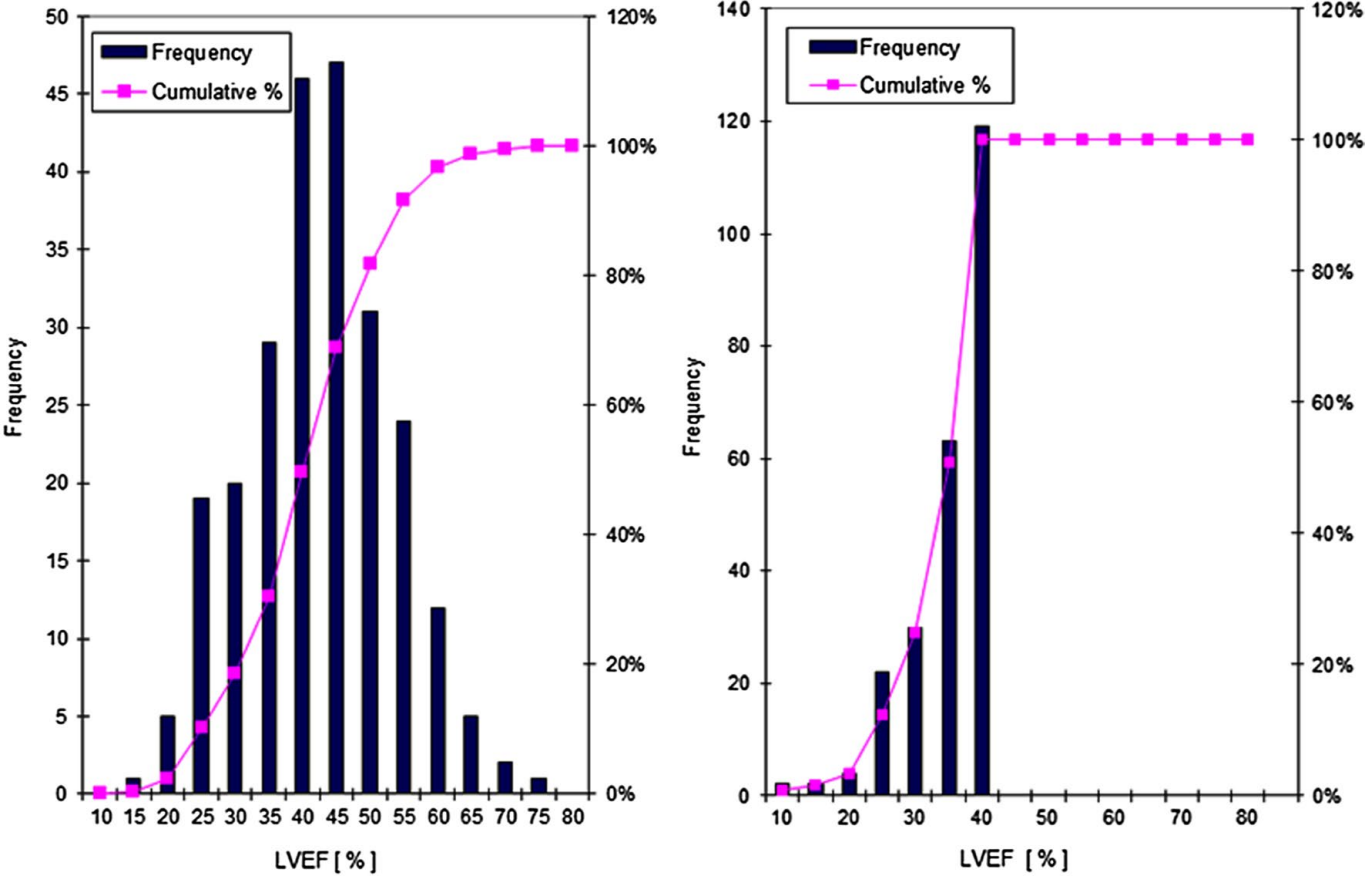
Histograms: EF values during the first and second. Truncated data at 40% on the occasion of the first visit due to inclusion criteria for groups 1 and 2, approximately Gaussian distribution during second visit. *LVEF* left ventricular ejection fraction

Biplane LVEF was determined by two-dimensional echocardiographic imaging according to Simpson [22], using the 3S probe of Vivid 7 (Vivid 7, GE Ving Med, Horten, Norway) was used. Means of three biplane planimetric measurements were rounded to multiples of 5%. Follow-up examinations were usually performed by a different physician according to the same protocol. However, follow-up LVEF were not rounded to a multiple of 5% but to integers. Follow-up observations included the complete range of LVEF values and were not restricted to patients with up to 40% LVEF (Fig. 1).

### Statistics

The descriptive statistics were means ± standard deviation (SD). Scattergrams were used to visualize change in LVEF. Patients were pre-selected according to baseline LVEF and LVEF values were subject to some measurement error and the extent of biological variation amongst patients was unknown. The changes during follow-up therefore tend to regress towards the mean (RTTM). In the selected population, patients who happened to reveal LVEF values that were lower than their long-term average are over-represented as compared to patients with correct or higher LVEF values. These patients tend to regress to their long-term mean with the result that the average LVEF increase is exaggerated over the unknown real improvement for physiological reasons.

In order to determine the extent of the selection bias, LVEF change was plotted against the time interval between baseline and follow-up determination. A Locally Weighted Regression Scatter Plot Smoothing (LOWESS) regression function was superimposed on the scattergram [25]. The point where this line intercepts with the ordinate can be interpreted as estimate of the selection bias since it can be assumed that with a time interval of zero no change in true LVEF can take place and the change observed is a mixture of measurement error and selection bias only.

## Results

The majority of patients were given a combination of ACE inhibitors/angiotensin receptor blockers (ARB) (85.8%), beta blockers (80.5%), statins (63.1%), aldosterone antagonists (23.8%) and diuretics (59.2%). Seven patients (2.5%) were lost to follow-up. Patients had received revascularisation by percutaneous intervention (PCI) in 74.3% and by coronary artery bypass graft surgery (CAGB) in 25.7%. Patients were scheduled for echocardiographic follow-up measurement of LVEF after mean of 441 ± 220 days.

There were 76 patients in group 1 (LVEF <30%) and 201 in group 2 (LVEF 30–40%) Baseline characteristics for the two strata and for the total group are listed in Table 1. In Group 1 80.3% of patients were males (mean age 66.1 ± 11 years), in group 2 84.6% were males (65.4 ± 10 years).

**Table 1:** Baseline characteristics

	Group 1	Group 2	Group 1 + 2
n (%)	76 (27.4)	201 (72.6)	277 (100)
Male, *n* (%)	61 (80.2)	170 (84.5)	231 (83)
Age (years, mean ± SD)	66.1 ± 11	65.4 ± 10	65.5 ± 10.6
Ejection fraction (%)	26.2 ± 4.8	38.2 ± 2.5	35.3 ± 6.1
Mean follow-up (days)	488 ± 203	414 ± 197	441 ± 220
Median time interval after MI (months)	1.55	1.2	1.3
ICD implanted (*n*)	27	6	33

Values are means (±standard deviation, SD), if not indicated otherwise

*MI* myocardial infarction, *ICD* implantable cardioverter defibrillator

**Fig. 2:**
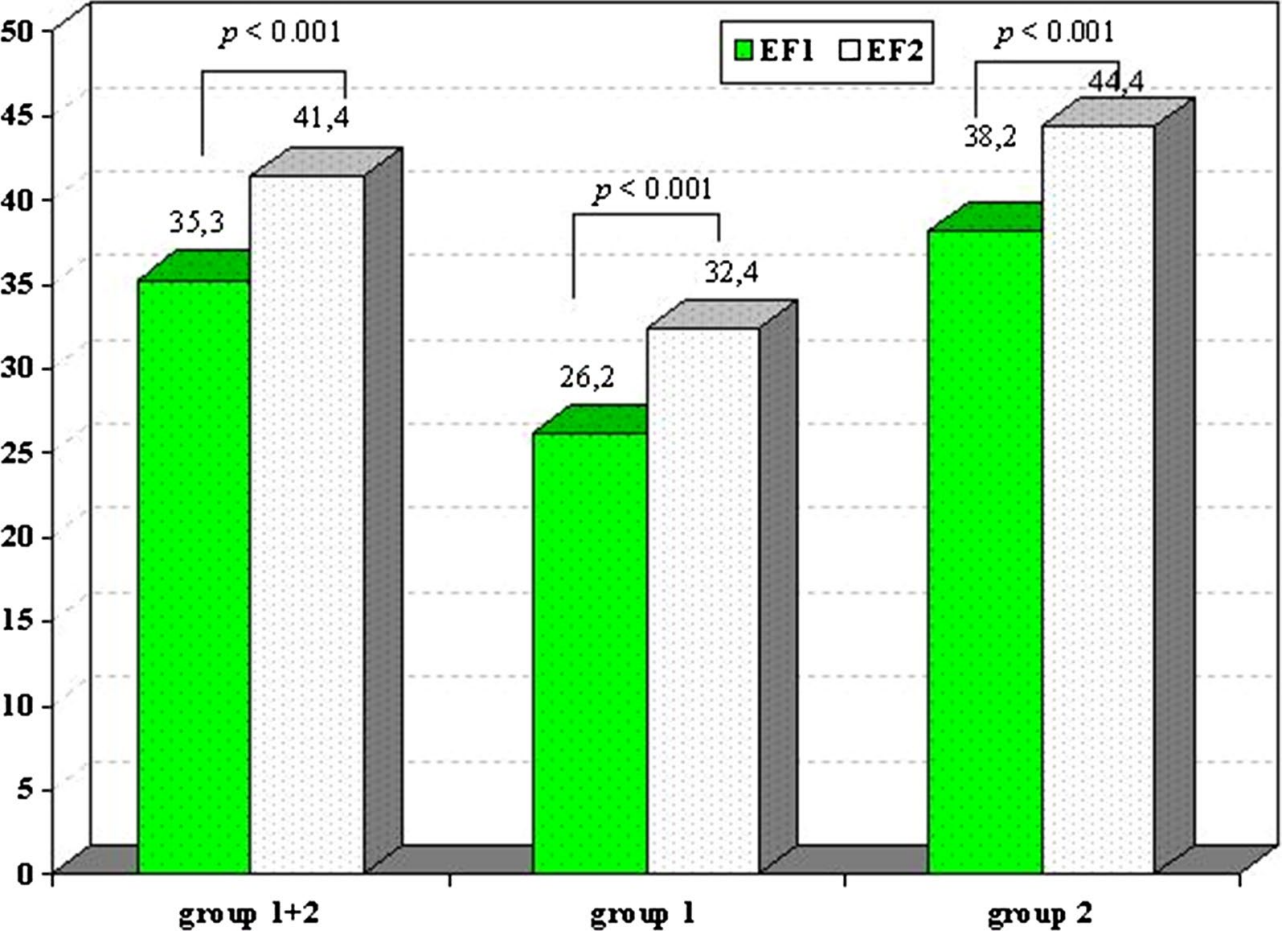
Change in left ventricular ejection fraction without bias correction. Significant increase of left ventricular ejection fraction for groups 1 and 2 as well as for the two groups considered separately. *EF* left ventricular ejection fraction

There was a significant increase of left ventricular ejection fraction for the total patient population of 6.2 ± 9.0% (35.3 ± 6.1% in group 1 vs. 41.4 ± 10.7 in group 2; *p* < 0.001). In both groups, the pattern of change was similar: in group 1, LVEF increased from 26.2 ± 4.8% to 32.4 ± 8.5% (*p* < 0.001). In group 2, there was also an improvement of LVEF from 38.2 ± 2.5 to 44.4 ± 9.6% (*p* < 0.001) (Fig. 2). In 167 (69%) patients, an improvement of LVEF was observed, in 33 patients (13.6%) LVEF remained constant, in 42 patients (17.4%) LVEF decreased. In 11 patients (18%) of group 1, LVEF increased to a value above 40%, in 19 patients (32%) to a value in the range of 30–40%.

**Fig. 3:**
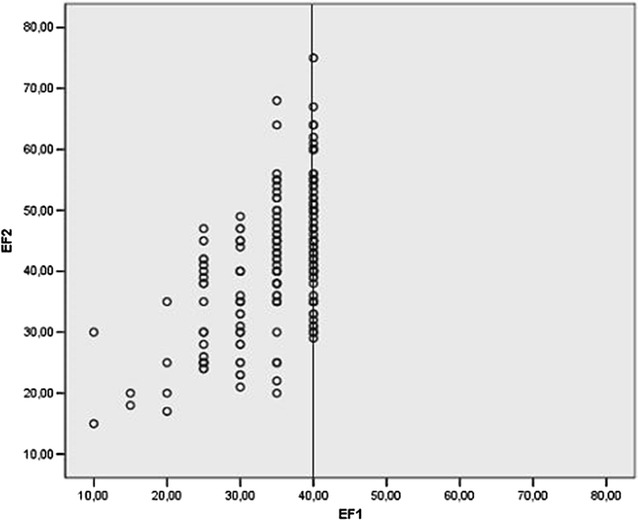
Scatter plot: relation between EF values during first (EF1) and second visit (EF2). Truncated data at 40% on the occasion of the first visit due to inclusion criteria for groups 1 and 2, approximately homogeneous during second visit. *EF* left ventricular ejection fraction

In the scattergram (Fig. 3) which shows both baseline and follow-up LVEF measurement of individual patients, there was a steady trend towards higher LVEF values, but with a considerable scatter due to measurement error and individual trend variability that cannot be distinguished from one another. In consequence of the inclusion criterion of LVEF <40%, the scattergram is limited by a vertical line to the right side.

**Fig. 4:**
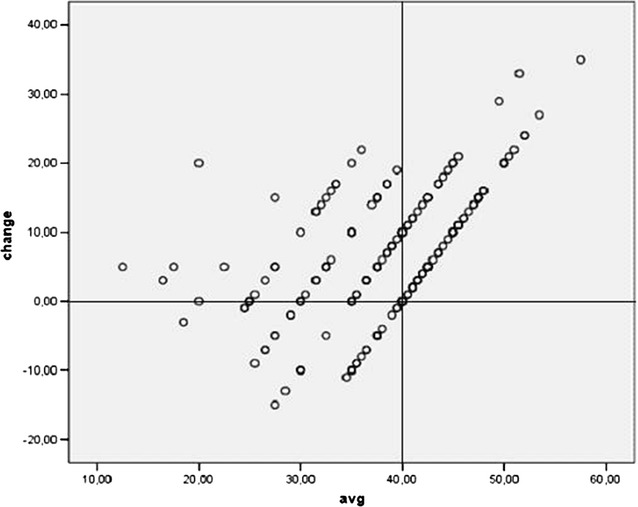
Scatter plot: axes of Fig. 3 are rotated by 45%. Differences of follow-up and baseline LVEF measurements (ordinate) are plotted vs. averages (abscissa) analogous to Bland–Altman plots. *avg* average

Compared to Fig. 3, in Fig. 4 the axes are rotated by 45%. Differences between follow-up and baseline LVEF measurements (ordinate) are plotted vs. averages (abscissa) analogous to Bland–Altman plots. If no patient selection had taken place, there were values in the right lower corner forming an ellipsoid scatter. The average differences are too high and were attributable to the experimental design; this demonstrates how regression towards the mean and the corresponding selection bias originate. If we restrict the analysis to patients with an average of less than 40% LVEF (n = 122), the average difference is 2.0% (95%-CI 0.55–3.5, p = 0.007). This figure estimates the average true LVEF change after correction for the selection bias.

**Fig. 5:**
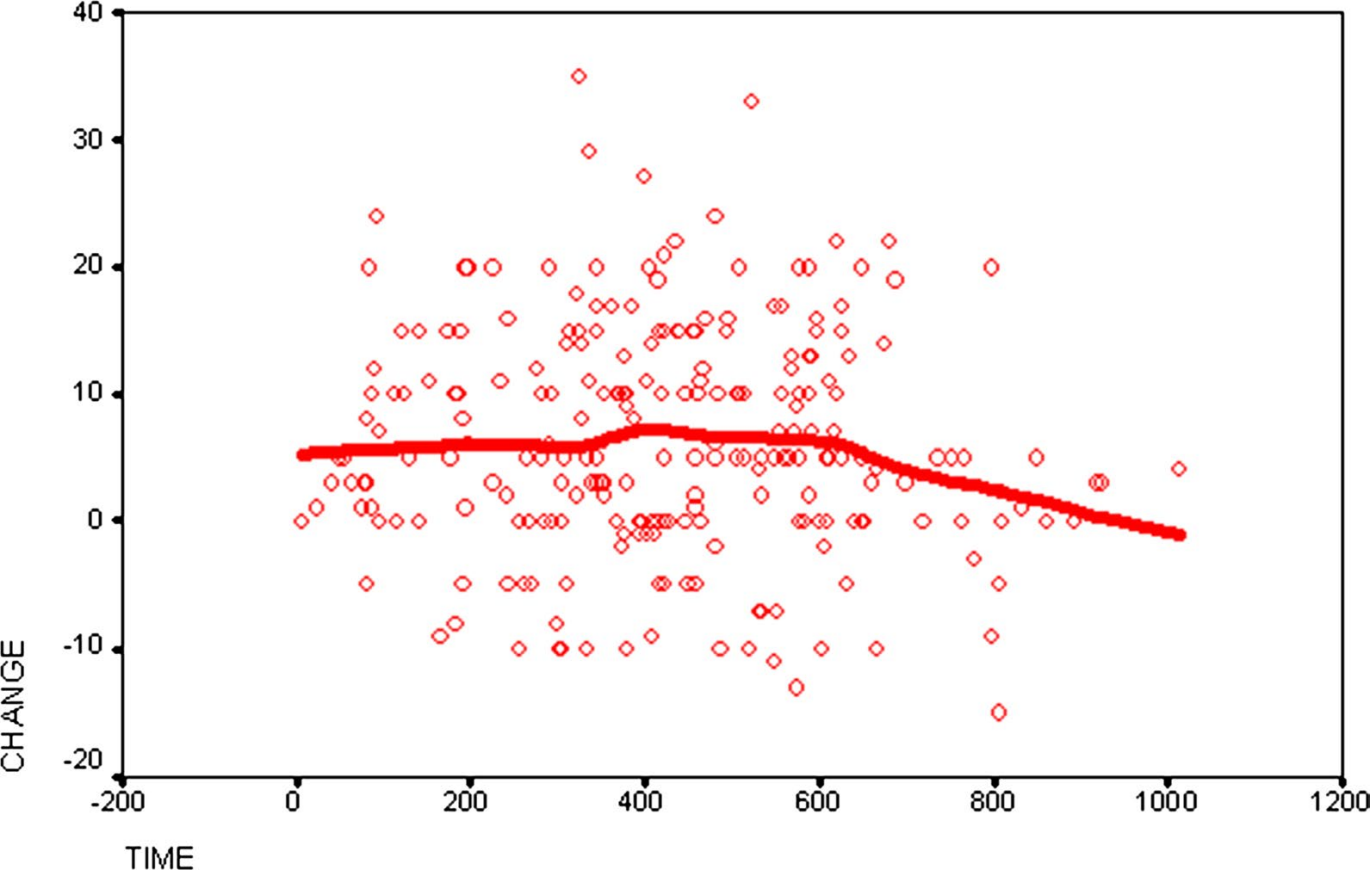
LOWESS regression analysis. Influence of time between first and second measurement and change in LVEF

Figure 5 contains the LOWESS line that demonstrates the time-dependence of the apparent improvement. The cutoff with the ordinate is at 7%. The LVEF increases by about 2% up to about 15 months and seems to deteriorate again after 20 months.

**Fig. 6:**
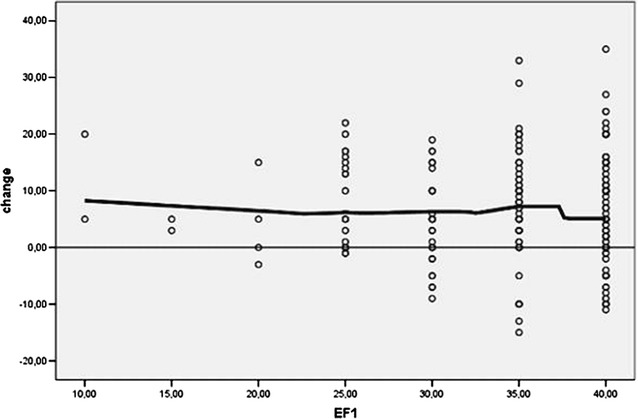
Change of LVEF does not depend on initial values (EF1). There was a homogeneous spread in the EF value changes. *EF* left ventricular ejection fraction

Likewise, the extent of change shows no dependence on the initial EF value (Fig. 6).

## Discussion

According to the present analysis, apparent improvements of LVEF to a considerable extent can be attributed a statistical phenomenon and are not true effects. Second, LVEF may show considerable intraindividual variation during follow-up. Owing to such high variability, a single measurement may not be reliable enough to justify a decision on ICD indication.

The increase in LVEF in the study population was moderate at 6%. This is in line with the expectation based on a pathophysiological viewpoint, that true left ventricular function it is highly improbable to recover to normal in patients with a damaged post-MI ventricle. This applies to patients with severely reduced LV function as well as patients with a moderate reduction. Although complete revascularization is one of the most important interventions for improving left ventricular function and reducing ischemic-driven ventricular tachyarrhythmias after MI, the recovery of impaired LVEF even after complete revascularization in post-myocardial patients usually is moderate at best. Recovery of systolic function in patients with ischemic cardiomyopathy can be expected only in stunned and hibernating myocardial segments [19]. In our data, after accounting for statistical artefacts, analysis of the time dependence of recovery reveals a slight increase by about 2% up to more than 1 year followed by a steady state and a slight decrease after almost 2 years, most likely due to progression of CHD.

The second important result is that the raw LVEF measurements suggest more marked changes in the preselected individuals. The change in mean values in subgroup populations is strongly affected by regression towards the mean (RTTM). Regression towards the mean reflects a statistical effect describing the relationship between two linked measurements [17–21]: if the initial value is above or below the mean, the later value is likely to be closer to the mean than the initial value. Studies of treatment effects in clinical trials and longitudinal follow-up investigations of disproportionate variables usually analyze whether there is a correlation between the change in the variable and its initial value, but the results are biased by the RTTM effect [26–29]. RTTM appears to be a selection phenomenon, emphasizing the need to include control groups in order to adjust for the bias caused by RTTM. Our study comprised a predefined subgroup of patients with LVEF of ≤40%. A second measurement allowed the complete range of possible LVEF to be established. Consequently, asymmetrically truncated subgroups were compared with a homogeneous Gaussian distribution of ejection fraction values. This entailed an overestimation of the rise in LVEF, which can only be avoided by excluding all patients with LVEF >40% in the second measurement. After taking RTTM into consideration, the improvement of LVEF was less impressive. Most importantly, it was not significant for clinical decisions, even though major proportion of patients had a mild increase of LVEF as measured after revascularization. Additionally, assessed improvement of LVEF is a combination of interindividual and intraindividual variability, RTTM and actual change of LV function. Variability should be taken into account when the ejection fraction is used as a parameter of improved or worsened cardiac function. Even in comparison of homogenous groups, individual changes of LVEF should be interpreted as significant only when they exceed the total variability of echocardiographic imaging [17, 24].

In normally distributed variables with homogeneous sample sizes, conventional comparison tests are appropriate to assess changes of values. However, the usual statistical methods for subgroup analyses should be avoided because of preselection bias and other measurements like score tests based on truncated data, regression based *t* test, likelihood tests or Wald´s test should be preferred.

## Limitations

Our study has a number of potential limitations. First, our patients displayed a wide range of time interval between MI and first assessment LVEF. Although the median was 1.2 months, there were several patients with a considerably longer interval, which at least in the infarctional area reflects a stable myocardial scar without remodeling capacity. Second, the initial values of left ventricular ejection fractions were rounded up or down to 5% steps, which include a maximum error of 2%. A third potential limitation stems from different observers in respect of the initial LVEF measurement. The second LVEF was assessed by only one investigator to limit interindividual variability as much as possible.

## Conclusions

Even after complete revascularization, left ventricular dysfunction in post-myocardial heart failure patients remains largely unchanged. Change in mean values in subgroup populations are influenced by regression toward the mean. However, since physicians have no other sources of information at hand, they are bound to interpret the observed changes as real improvements and thus may tend to draw excessively optimistic conclusions with regard to the clinical course of their patients. While the real (as opposed to the apparent) recovery of LVEF after early post-MI revascularization turned out to be rather moderate, a single LVEF measurement is probably not reliable enough to allow a definite decision on ICD implantation. In consequence of the considerable instability of LVEF determinations that was observed, LV function should be assessed repeatedly before making treatment decisions with long-term effects such as ICD implantation for primary prevention of SCD.

## Abbreviations

CABG: coronary artery bypass graft operation
ICD: implantable cardioverter-defibrillator
LOWESS: locally weighted regression scatter plot smoothing
LVEF: left ventricular ejection fraction
MI: myocardial infarction
PCI: percutaneous coronary intervention
PreSCD II: prevention of sudden cardiac death ii
RTTM: regression toward the mean
SCD: sudden cardiac death
SD: standard deviation
